# A new cascade of HIV care for the era of “treat all”

**DOI:** 10.1371/journal.pmed.1002268

**Published:** 2017-04-11

**Authors:** Matthew P. Fox, Sydney Rosen

**Affiliations:** 1 Department of Global Health, Boston University School of Public Health, Boston, Massachusetts, United States of America; 2 Department of Epidemiology, Boston University School of Public Health, Boston, Massachusetts, United States of America; 3 Health Economics and Epidemiology Research Office, Department of Internal Medicine, School of Clinical Medicine, Faculty of Health Sciences, University of the Witwatersrand, Johannesburg, South Africa

## Abstract

Matthew Fox and Sydney Rosen discuss a cascade of HIV care adapted to WHO-recommended antiretroviral therapy irrespective of CD4 cell count.

**Summary points**

- Now that the World Health Organization has recommended antiretroviral therapy for all people infected with HIV regardless of their CD4 count, millions more people will become eligible for HIV treatment.
- We and others have previously described the cascade of care for HIV treatment for monitoring retention in care and targeting interventions to improve retention to areas of greatest need.
- To inform research and track progress, we propose a new cascade for the “treat all” policy that effectively removes the pretreatment waiting period.
- Current data on retention through each stage of care will have limited value under this new cascade, as some stages will no longer exist while others will include patients who were previously not eligible.
- New data are needed to parameterize the care cascade under the “treat all” policy, and new interventions will be needed to improve retention across the new cascade.

## Introduction

Over the past decade, the concept of the “cascade of care” has become a standard way of describing and analyzing patient behavior in the interval between an HIV diagnosis and long-term retention on antiretroviral therapy (ART) [1,2]. Care cascade research has helped quantify losses of patients from care, identify the points of greatest attrition, and target interventions to addresses losses [2,3].

Most cascade research focuses either on the period after starting ART or between HIV testing and ART initiation (i.e., “pre-ART care”). Pre-ART care is focused on immunologic (CD4 count) monitoring for patients known to be HIV-infected but not yet ART eligible. In 2011, we reviewed retention in pre-ART care in sub-Saharan Africa and revealed disturbingly high attrition at every point in the pre-ART cascade [2]. We defined three stages of pre-ART care and reported that large proportions of patients who had HIV tests did not return for CD4 results or clinical staging (Stage 1), did not remain in care until treatment eligibility (Stage 2), or did not initiate ART if eligible (Stage 3). Retention was particularly poor in Stage 2, in which only 55% (range 42%–95%) of patients were retained in pre-ART care. Later reviews have confirmed these findings [4–6].

In 2015, the World Health Organization (WHO) recommended ART be offered to all HIV-positive individuals regardless of CD4 count, ushering in the era of “treat all” [7]. Among the benefits expected by WHO are “significant increases in ART uptake and linkage to care, reduction in the time between HIV diagnosis and ART initiation regardless of baseline CD4 cell count and an increase in the median CD4 value at ART initiation” [7]. UNAIDS has adopted the 90–90–90 targets (90% of those infected knowing their HIV status, 90% of those diagnosed on treatment, and 90% of those on treatment virally suppressed), which use the care cascade in order to track progress and end the AIDS epidemic [8].

One immediate implication of the guideline change is the effective elimination of Stage 2 of pre-ART care, which served as a holding place for patients not yet ART eligible. The challenge facing health care providers will shift from retaining patients in Stage 2 to improving Stage 1 (getting patients into care after an HIV diagnosis, or “linkage to care”) and Stage 3 (ART initiation). Stages 1 and 3 will remain essential, with the ultimate goal of creating a smooth transition from testing to treatment. A new framework for understanding, implementing, and monitoring the HIV care cascade is thus needed.

## Implications of the new guidelines for the current HIV care cascade

We previously divided pre-ART care into three stages (S1 Fig). Others have presented variations on this pre-ART model, often including a fourth stage for retention on ART. As noted, attrition is high at every stage; Box 1 summarizes existing estimates. The new WHO guidelines have implications for each stage of care.

**Box 1. Stages in the previous cascade of care**

Stage 1: Testing to staging and/or receipt of CD4 results

In our 2011 review, we estimated the median proportion of patients completing Stage 1 at 59% (range 35%–88%) [2], a finding confirmed in a follow-on review by others a year later that reported a rate of 57% (95% CI: 48 to 66%) [5]. Estimates of Stage 1 are hindered by the lack of data systems that track patients from testing to care, making the total number of diagnosed individuals who could be linked to care difficult to determine.

Stage 2: Staging to ART eligibility

Our 2011 review [2] found a median of 55% (range 42%–95%) of patients completing this stage; the 2012 review mentioned above reported 45% [5]. Stage 2 is the most difficult to evaluate because it requires lengthy follow-up and because there is substantial heterogeneity in services offered and in the timing of recommended follow-up CD4 assessments.

Stage 3: Eligibility to ART initiation

We estimated that a median of 68% (range 14%–84%) [2] of patients completed this stage, again very similar to the 66% (95% CI: 58 to 73%) reported in the follow-on review [5]. Within the cascade, Stage 3 can be evaluated most readily because it has clear starting and ending points that are expected to be close together in time and occur at the same treatment site for most patients.

Stage 4: Retention on treatment

We recently estimated adult retention on treatment globally at 94% and 83% at 6 and 12 months, respectively [9]. Within sub-Saharan Africa and among children, the averages were similar [10]. While these estimates were fairly precise, they may either over- or understate actual retention. Much of the data on retention come from well-resourced clinics that have better retention than national averages. On the other hand, the inability to track patients who move between clinics, the so-called “silent transfers” [11], leads many studies to overestimate losses to care.

In that same review, we estimated long-term retention on ART and found that adult retention globally was 74%, 68%, 64%, and 60% at 24, 36, 48, and 60 months, respectively [9]. Estimates within sub-Saharan Africa and among children were similar [10]. Retention after the first 12 months appears to decline at a much slower rate than the rate that occurs over the first year on treatment.

### Stage 1: Testing to enrollment in care

Stage 1 covers the interval from receiving an HIV-positive diagnosis to receiving CD4 count results or learning ART eligibility. Patients may be lost in Stage 1 between having an HIV test and enrolling in care or, if tested at a clinic, between providing a CD4 count blood sample and returning for results. Under the new guidelines, CD4 counts will no longer be required to establish ART eligibility. However, patients must still enroll in care after diagnosis. Home- and community-based strategies have been proposed to reduce loss from this stage [12,13], but these models are not currently being employed at scale, and most patients only receive services once they enroll at a clinic.

Existing data on Stage 1 describe the behavior of patients asked to enroll in care without knowing whether they would be offered ART. A disease progression threshold for treatment eligibility has existed since the launch of national treatment programs in 2004. The behavior of patients who tested positive for HIV in the past, which determined the proportion who enrolled in care after a positive test, may therefore not predict the behavior we will see under a “treat all” policy.

The effect of the new guidelines on linkage to care could be positive or negative. Knowing all patients who enroll in care will be offered treatment may encourage enrollment for those previously deterred from visiting a clinic because of the belief that nothing would be done—and could also increase willingness to have an HIV test in the first place. Conversely, for those not ready, motivated, or empowered to accept the burden of lifelong treatment, that same knowledge may discourage engagement with the health care system. Monitoring linkage to care, which requires a denominator of all those who test positive, will be essential to assessing the effect of the new guidelines on Stage 1.

### Stage 2: Retention in pre-ART care until treatment eligibility

Stage 2 covers the interval from enrollment to ART eligibility, when patients who are not yet eligible for ART must make regular monitoring visits until they meet the prevailing eligibility criteria. Under a “treat all” policy, Stage 2 will cease to exist. A few patients will still require a delay in initiating ART—for example, for tuberculosis treatment—but even for those delayed, because this period will be sufficiently compressed, a separate stage is no longer needed in the cascade. For the vast majority of patients, linkage to care and ART initiation should be synonymous, and research on retaining patients in pre-ART monitoring should no longer be needed.

### Stage 3: Eligibility to treatment initiation

Stage 3 of the current cascade lasts from determination of eligibility to ART initiation. Losses in this stage likely relate both to health system barriers, such as requirements for multiple clinic visits and delays in receiving laboratory test results [14], and to patient factors such as time constraints or reluctance to commit to lifelong treatment. Interventions to accelerate this stage of care have shown promise [15–17], but attrition between eligibility and treatment initiation is likely to persist.

Under a “treat all” policy, the characteristics of patients entering Stage 3 should change substantially, with an increasing proportion of asymptomatic patients who face different barriers to initiation. Removing the eligibility threshold can be expected to increase initiation overall, but whether this change will improve Stage 3 retention is hard to predict. If the other obstacles to initiating ART remain unchanged—or worsen, as increasing patient volumes further strain already overburdened health care systems [18]—many patients will continue to be lost from care before their first dose of medications is dispensed. Monitoring treatment initiation rates and timing will thus remain essential to assessing the effect of the new guidelines on Stage 3.

### Stage 4: Retention on ART

Though not included in S1 Fig, Stage 4—retention after starting ART—remains a critical part of the cascade. Although the new guidelines will not directly affect Stage 4, patients entering this stage may differ from those in the past. Baseline CD4 counts have been increasing in recent years [19], and this will likely persist under a “treat all” policy, creating a less-immunocompromised population beginning Stage 4 than in the past. At the same time, the new guidelines pose several risks for long-term retention. It is likely the new guidelines will simply shift some attrition from before to after treatment initiation (i.e., from current Stages 1–3 to Stage 4) [20], as was observed in our recent study of same-day treatment initiation [16]. Patients starting treatment early in disease progression, without ever having developed symptoms, may have different adherence patterns than those treated until now. For these reasons, existing data on retention on ART may not predict future outcomes, and monitoring of retention will remain as important as ever.

While early retention on ART is marked by losses due to early death (largely related to late presentation) and high rates of loss to care, later retention on ART (after the first year) is less dramatic, with mortality stabilizing and losses diminishing. While there is little published evidence, we speculate that many of the losses from individual clinics after the first year are in fact undeclared transfers to new treatment sites. Later retention is thus unlikely to be affected by the guideline changes.

## A new HIV care and treatment cascade

**Fig 1:**
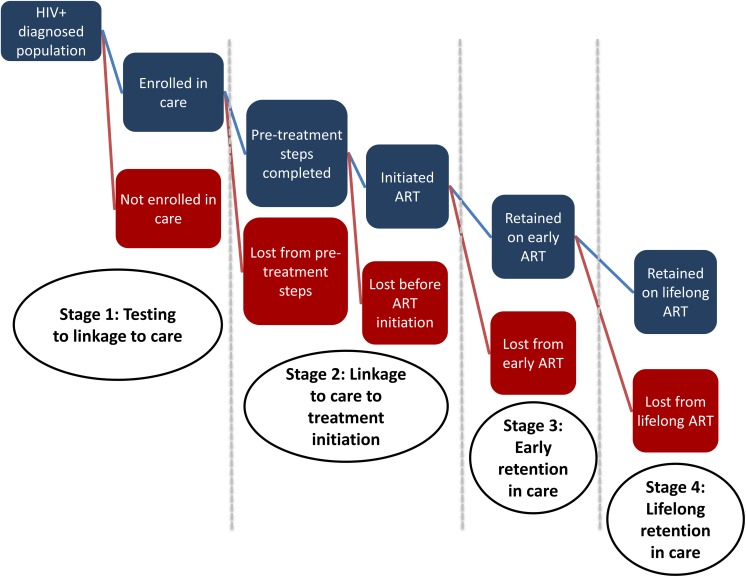
A New HIV Care Cascade and Opportunities to Become Lost to Care After Moving to Treatment for All

As this discussion suggests, a new model will be needed to capture the delivery of HIV care and treatment under a “treat all” policy. In Fig 1, we present a new cascade, with the aim of more effectively monitoring the outcomes as guidelines evolve. This cascade, as in previous cascades, begins after patients are diagnosed with HIV. HIV testing could certainly be added as the starting point, however, as some have suggested [21]. As the cascade is meant to capture patient care-seeking behavior and not the results of treatment, it also does not explicitly include adherence, viral suppression, or mortality (either on or off treatment). These could be added as subcategories when the cascade is used for specific purposes. This new cascade diverges from S1 Fig in three ways. First, we have eliminated the previous Stage 2 so that the pre-ART period now has only two stages: HIV testing to linkage to care (Stage 1) and linkage to care to ART initiation (Stage 2). Second, to reflect the differences in retention on ART between the first and later years, we have divided the previous Stage 4 into two new stages: Stage 3 for early retention and Stage 4 for later lifelong retention. Finally, determination of treatment eligibility, previously included in Stage 1 and as the transition from Stage 2 to Stage 3, is no longer required under a “treat all” policy. In the new cascade, we define linkage to care (or enrollment in care) as the point at which an HIV-infected individual is recorded as making a first visit to a health care facility or other ART provider. In the new cascade, all patients completing Stage 1 immediately enter Stage 2. The challenge for health care providers will be to make this transition as seamless in practice as it appears in the illustration. WHO recommends differentiated care (i.e., care delivered in differing locations, frequency, intensity, etc. for those in different stages of illness, with different levels of adherence to treatment, etc.) as part of the process of ensuring better retention in care across the cascade, and the new consolidated guidelines for “treat all” make specific recommendations [7]. The guidelines also provide a summary of the evidence base for interventions to improve the care cascade.

## Implications for research

The new “treat all” cascade guides us towards two points of intervention that appear to be most important for improving outcomes. The first is linkage to care. We recently reviewed the literature on pre-ART interventions and found few examples of successful efforts to increase the rate at which patients diagnosed with HIV enroll in care [22]. As the new guidelines are likely to alter the population tested for HIV, and not just treatment eligibility, research to strengthen linkage to care is urgently needed.

The second priority point for intervention is treatment initiation. Under current practice, starting treatment can take multiple clinic visits spread over several weeks [23,24], and patients can take weeks and several visits between treatment eligibility and ART initiation. Accelerating treatment initiation by compressing the steps needed to initiate ART has demonstrated that nearly all eligible patients can be initiated on treatment if health systems are designed to do it [15–17]. As with linkage to care, the population presenting for treatment initiation is likely to change under a “treat all” policy, making it imperative that more efficient models of initiation be developed. While we do not know what retention will look like for patients with high CD4 counts who were not previously eligible, previous work with women in prevention of mother-to-child transmission programs suggests it may not be as high as for those who initiate because of their own health condition [25,26].

The new cascade also has implications for research design. In particular, it emphasizes the importance of reporting treatment initiation, if not early retention on ART, as an endpoint for pre-ART evaluations rather than the intermediate outcome of linkage. Under a “treat all” policy, linkage to care and treatment initiation are intended to be synonymous—once enrolled in care, all patients should start ART. An intervention that increases linkage but not the number of patients initiating ART or results in higher than expected attrition shortly after ART initiation may not be as effective as it would have appeared had linkage to care been the only outcome reported. At the same time, the new cascade highlights the importance of distinguishing reporting between rates and absolute numbers. An intervention that increases linkage to care will increase the numbers of patients presenting for ART initiation, leading to larger numbers initiating ART even if it does not change the initiation rate. Future studies should seek to describe the full impact of interventions on the cascade, at least up to the point of treatment initiation and ideally through early retention on ART. The cascade also has implications for the data systems needed to monitor progress in each stage of care. These will differ by stage but would include records of HIV testing and patient linkage data for Stage 1 and patient data and vital statistics for Stages 2–4.

This new cascade, like previous cascades, is depicted as linear in nature—patients enter and proceed through stages of the cascade or drop out, never to return. In fact, patients can cycle in and out of care, leaving at one stage (e.g., long-term retention) and reentering at another (e.g., initiating ART)—what some have called the side door in the cascade [27]. We anticipate that users of the new cascade will adapt it to the situations they face, which may include greater or lesser degrees of cycling in and out at different points in the cascade. Cycling of patients is also important for monitoring progress towards global targets, as it is not clear whether to consider a patient who returns to care after a gap (i.e., “cycles”) a “success” or “failure” for assigning outcomes. This may ultimately depend on the specific research question being asked.

WHO reports that by late 2016, it anticipated that one-third of all low- and middle-income countries had adopted “treat all” as national policy, with more anticipated by the end of the year [28]. Forward-looking research, however, will increasingly focus on how to deliver services once all patients are eligible for ART, regardless of disease stage. This care cascade engendered by the new guidelines is simpler to model than the previous approach, and we anticipate it will also be simpler to evaluate once in use. The hope now is that it will also be both simpler and more effective in practice.

## Supporting information

S1 Fig The previous HIV care cascade and opportunities to become lost to care under earlier guidelines. (TIF)
