# Supplementary figures and images for: A new cascade of HIV care for the era of “treat all”
Source: PLoS Med. 2017 Apr 11;14(4):e1002268. doi: 10.1371/journal.pmed.1002268 (PMC5388465; doi:10.1371/journal.pmed.1002268)

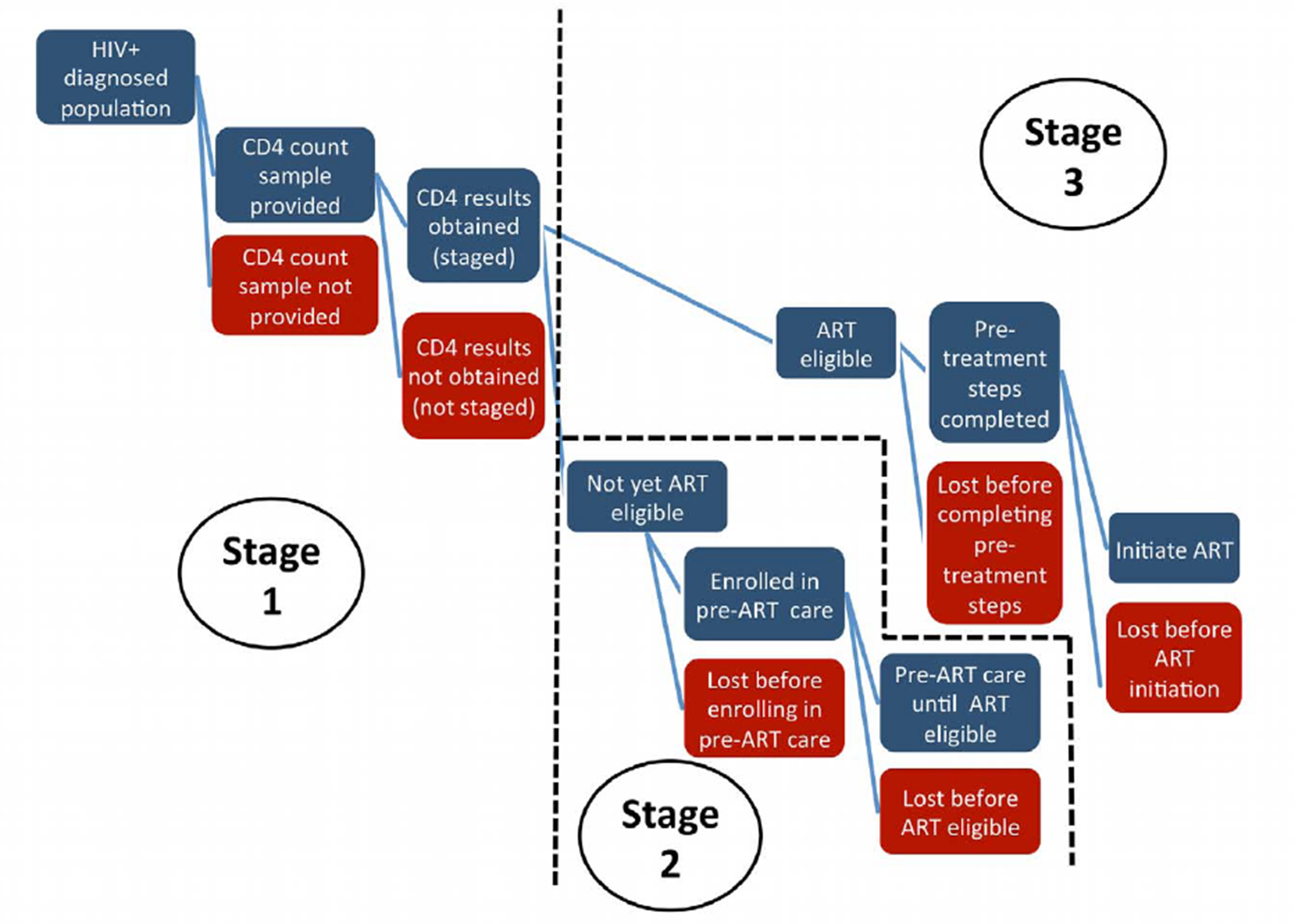

Supplement: S1 Fig — (TIF) [file pmed.1002268.s001.tif]
